# Adsorption and Corrosion Inhibition Studies of Some Selected Dyes as Corrosion Inhibitors for Mild Steel in Acidic Medium: Gravimetric, Electrochemical, Quantum Chemical Studies and Synergistic Effect with Iodide Ions

**DOI:** 10.3390/molecules200916004

**Published:** 2015-09-02

**Authors:** Thabo Peme, Lukman O. Olasunkanmi, Indra Bahadur, Abolanle S. Adekunle, Mwadham M. Kabanda, Eno E. Ebenso

**Affiliations:** 1 Department of Chemistry, School of Mathematical & Physical Sciences, North-West University (Mafikeng Campus), Private Bag X2046, Mmabatho 2735, South Africa; E-Mails: pemethabo@yahoo.com (T.P.); waleolasunkanmi@gmail.com (L.O.O.); bahadur.indra@gmail.com (I.B.); sadekpreto@gmail.com (A.S.A.); Mwadham.Kabanda@nwu.ac.za (M.M.K.); 2 Material Science Innovation & Modelling (MaSIM) Research Focus Area, Faculty of Agriculture, Science and Technology, North-West University (Mafikeng Campus), Private Bag X2046, Mmabatho 2735, South Africa; 3 Department of Chemistry, Faculty of Science, Obafemi Awolowo University, Ile-Ife 220005, Nigeria

**Keywords:** corrosion inhibition, polarization, quantum chemical calculation, adsorption, synergistic interaction

## Abstract

The corrosion inhibition properties of some organic dyes, namely Sunset Yellow (SS), Amaranth (AM), Allura Red (AR), Tartrazine (TZ) and Fast Green (FG), for mild steel corrosion in 0.5 M HCl solution, were investigated using gravimetric, potentiodynamic polarization techniques and quantum chemical calculations. The results showed that the studied dyes are good corrosion inhibitors with enhanced inhibition efficiencies. The inhibition efficiency of all the studied dyes increases with increase in concentration, and decreases with increase in temperature. The results showed that the inhibition efficiency of the dyes increases in the presence of KI due to synergistic interactions of the dye molecules with iodide (I^−^) ions. Potentiodynamic polarization results revealed that the studied dyes are mixed-type inhibitors both in the absence and presence of KI. The adsorption of the studied dyes on mild steel surface, with and without KI, obeys the Langmuir adsorption isotherm and involves physical adsorption mechanism. Quantum chemical calculations revealed that the most likely sites in the dye molecules for interactions with mild steel are the S, O, and N heteroatoms.

## 1. Introduction

Mild steel is a popular alloy of iron with many industrial applications. It is characterized with high mechanical strength and relatively low cost compared to other metal alloys. However, mild steel is highly susceptible to corrosion and the corrosion of mild steel has been a matter of great concern to various industries. Mineral acids, particularly hydrochloric acid, are frequently used in industrial processes such as acid cleaning, acid pickling, acid descaling, and oil well acidizing [1,2,3,4]. These acids constitute strong corrosive environments for mild steel and as a result, the study of the prevention of steel corrosion is always a subject of high theoretical and practical interest. The use of inhibitors has been identified as a convenient and cheap means of combating steel corrosion [5,6]. The inhibitors influence the kinetics of the electrochemical reactions which constitute the corrosion process, either by reducing the rate of metal dissolution in the corrosive medium and/or the cathodic reduction reaction. Corrosion inhibitors adsorb on the metal surface and thereby change the structure of electrical double layer. Most of the efficient inhibitors used in industry are organic compounds that contain oxygen, sulphur, nitrogen atoms, π-bonds, and/or aromatic ring(s) in their molecules. These electronegative atoms and functional groups have been reported to facilitate the adsorption of the inhibitors on metal surface [1,2,3,4,5,6]. One important factor to be considered in selecting a suitable corrosion inhibitor is non-toxicity, which many of the existing organic/inorganic corrosion inhibitors do not satisfy. Due to the increasing awareness of the use of environmentally benign chemicals in the industries and the stricter environmental protection regulations, the search for non-toxic efficient corrosion inhibitors is on the increase [7,8,9,10,11,12,13,14].

Organic dyes are compounds with promising corrosion inhibition characteristics. The presence of π-electron systems and heteroatoms in their molecules which is responsible for their colouration also suggests their high propensity to adsorb on metal surface and other adsorbents. There are different kinds of dyes and over a hundred thousand of dyes are commercially available [15,16]. About a million tons of different kinds of dyes are produced annually because of their important applications in various industries including textile, papers, additives, foodstuffs, cosmetic, leathers, agrochemical and pharmaceutical industries [17,18,19]. A number of studies have also been reported on the use of organic dyes as corrosion inhibitors [20,21,22,23,24,25,26,27]. The corrosion inhibition potentials of organic compounds depend on a number of factors such as the nature of the metal, the aggressive solution, molecular and electronic structure of the inhibitor, solubility and concentration of the inhibitor, pH and temperature. The molecular and electronic geometry play an important role in the trend of inhibition efficiencies of compounds of the same family under the same conditions. Organic molecules with planar molecular geometry often exhibit higher inhibition efficiencies than their less or non-planar counterparts [27]. Meanwhile, halide ions have been reportedly used to enhance the inhibition efficiency of organic compounds in some instances. Inhibitors with low solubility and those that are marked with possible concerned level of toxicity when used at higher concentrations are often utilized in small quantities in combination with halide ions in solutions. These molecules undergo synergistic interactions with halide ions, which lead to increase in their inhibition efficiency. However, antagonistic interactions between organic corrosion inhibitors and halide ions, leading to decrease in inhibition efficiency have also been reported in some cases [28,29]. The synergistic effect of halide ions on the corrosion inhibition potentials of organic molecules depend on the size of the ion, the electrostatic field set up by the charge of the ions on adsorption sites and the concentration of the halide ions. In this regard, previous reports on synergistic interactions of halide ions and inhibitor molecules have established that iodide (I^−^) ions give the best synergistic effects. As a result, recent findings on the improvement of inhibition efficiency via synergistic interactions with halide ions have focused mainly on the use of I^−^ ions.

Despite the popularity and wide applications of organic dyes, reports on the use of some of them, especially food colorants as corrosion inhibitors in aqueous mineral acids, are still scanty and dispersed. The inhibition effect of fast green (FG) on mild steel corrosion in 0.5 M HCl has been investigated using weight-loss and electrochemical methods at 300 K [30]. Fast red (FR), tartrazine (TZ), carmosine (CM), sunset yellow (SS) and amaranth (AM) have been used as corrosion inhibitors for aluminium-copper alloy in trichloroacetic acid. Their inhibition efficiencies were found to increase with increasing concentration of the colourants and the order of inhibition potentials was found to be FR < TZ < CM < SS < AM [31]. However, in another report, TZ, SS and AM promote the corrosion of copper in lactic acid with the order of corrosivity being AM < SS < TZ [32].

A comprehensive and comparative study of the corrosion inhibition properties of SS, AM, TZ, AR and FG on mild steel in hydrochloric acid using the combination of weight loss method, potentiodynamic polarization technique and quantum chemical calculations has not been reported. Corrosion inhibition properties of these food colourants in the presence of I^−^ ions and the effect of temperature on their inhibition potentials have not been investigated. These dyes have the tendency of forming lyotropic liquid crystals and dye aggregates, depending on the medium, concentrations and temperatures. These characteristics have been reported for SS [17,33,34,35] and may have effect on the corrosion inhibition properties of such dyes. However, since studies that investigate the corrosion inhibition properties of these food dyes at various concentrations and temperatures are not common, there has not been any report on the possible effect of their aggregation characteristics on corrosion inhibition properties.

**Figure 1:**
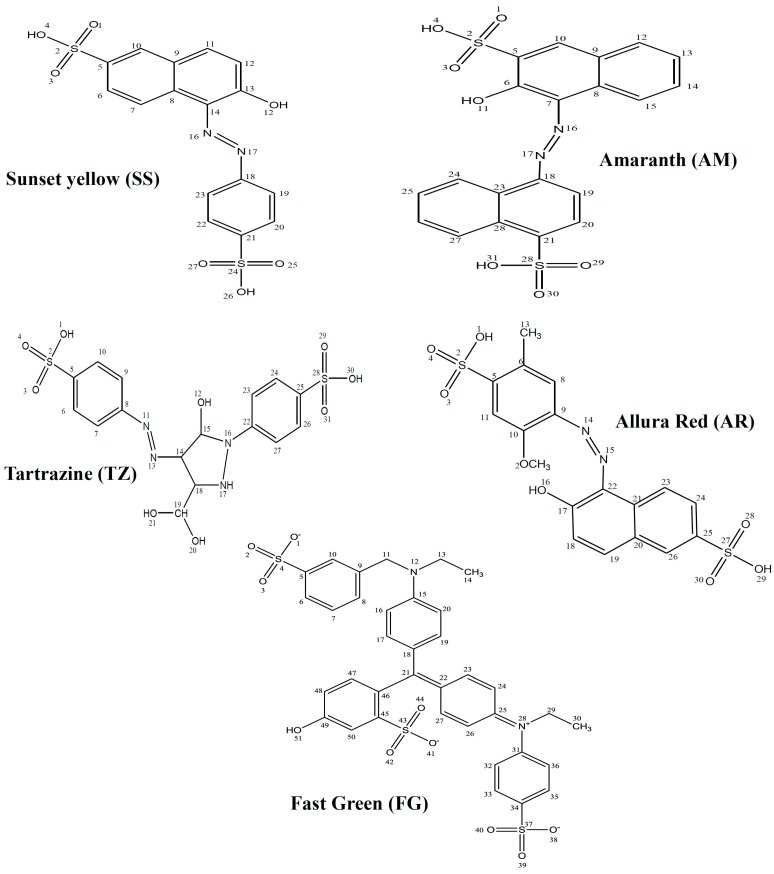
Molecular structures of the studied dyes.

The present study is in furtherance of our ongoing research on the design and investigation of non-toxic organic compounds as corrosion inhibitors. With the exception of AM, the set of dyes used in this work are among the permitted dyes used as food, drug and/or cosmetics colourants according to the Federal Food, Drug and Cosmetic Act of the United States of America [36]. The aim of this work is to investigate the corrosion inhibition properties of five organic dyes namely Sunset Yellow (SS), Amaranth (AM), Allura Red (AR), Tartrazine (TZ) and Fast Green (FG) on mild steel in 0.5 M HCl using gravimetric method, potentiodynamic polarization technique and quantum chemical calculations. Since most of the previous works on corrosion inhibition studies on dyes did not relate experimental and quantum chemical results, we have included the correlations of quantum chemically derived molecular and electronic parameters with experimental results in the present work in order to provide better explanations for the adsorption and inhibition behaviour of the studied dyes at molecular levels. The effect of synergistic interactions of I^−^ ions on the inhibition potentials of the dyes were also investigated. The molecular structures of the studied dyes are shown in Figure 1.

## 2. Results and Discussion

### 2.1. Gravimetric Method

The gravimetric measurements were carried out on mild steel immersed in 0.5 M HCl for 12 h in the absence and presence of various concentrations of the studied dyes. The experiments were conducted without and with addition of KI to the studied inhibitors at various temperatures.

#### 2.1.1. Effect of the Inhibitor Concentration and Temperature

**Figure 2:**
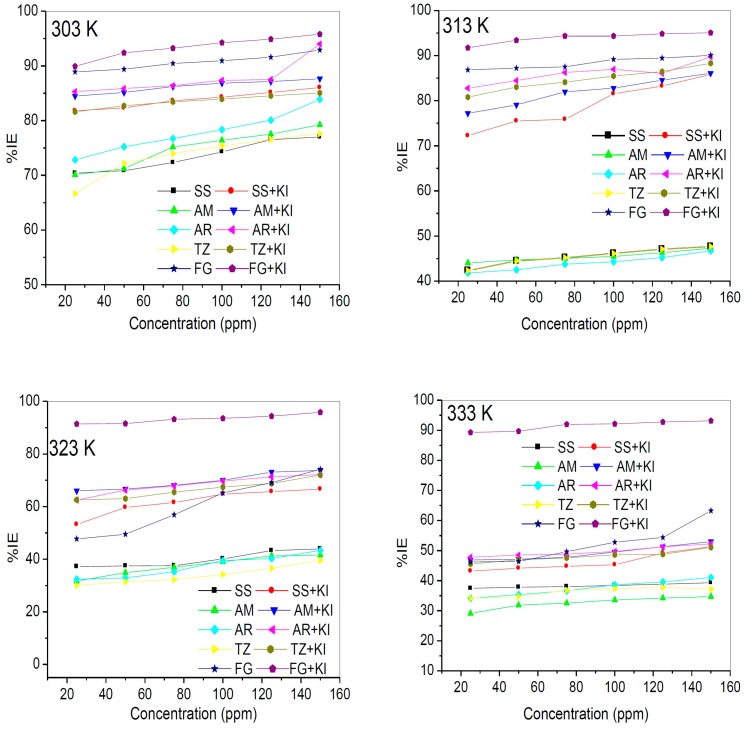
Inhibition efficiency against concentrations for the studied dyes without KI and with KI at 303–333 K.

The percentage inhibition efficiency (% IE_WL_) was calculated for the studied compounds at various concentrations and temperatures. The plots % IE_WL_ against concentrations of the inhibitors without and with KI at different temperatures are shown in Figure 2. The results show that the % IE_WL_ increases with increasing concentration and decreases with increase in temperature for all the studied dyes. The % IE_WL_ values in the presence of KI are generally higher than those without KI, which implies that the iodide (I^−^) ions undergo synergistic interactions with the inhibitor molecules thereby enhance the inhibition potentials of the compounds. The results in Figure 2 also reveal that at 150 ppm inhibitors concentration and 303 K, the trend of % IE_WL_ is such that FG > AR > AM > TZ > SS. With the % IE_WL_ values of TZ and SS being very close at 125 and 150 ppm (at 303 K), it could be inferred that this trend is similar to what was reported in literature for TZ, SS and AM as inhibitors for aluminium-copper alloy corrosion in trichloroacetic acid [31].

One apparent feature in Figure 2 is the variation in the trend of % IE_WL_ for the studied dyes at lower and higher concentrations. For instance, AM and SS have nearly the same values of % IE_WL_ at 25 and 50 ppm (at 303 K), but AM show significantly higher inhibition potentials than SS at 75–150 ppm at the same temperature. Similar observation was noticed between TZ and SS such that TZ shows lower % IE_WL_ value compared to SS at 25 ppm, significantly higher % IE_WL_ values than SS at 50–100 ppm but slightly higher % IE_WL_ values than SS at 125–150 ppm, all at 303 K. This observation also reflects at other temperatures. These dissenting trends at lower and higher concentrations may be due to different degrees of dye aggregation that could be undergone by the molecules of the dyes. It has been reported that organic compounds such as surfactants, ionic liquids, macrocylic compounds, quaternary ammonium salts, dyes, and Schiff bases with separated charges can undergo aggregations depending on concentrations, temperatures and other factors [4,17,33,34,37,38,39,40,41,42,43,44,45,46], and such aggregate interactions affect their inhibition efficiency [4,40,42].

The effect of KI on the inhibition potentials of the studied compounds is shown in Figure 2. Though all the dyes show higher values of % IE_WL_ in the presence of KI, the magnitude of the effect of KI varies with temperature and concentration of the inhibitors. At 303 K, the synergistic interactions of the dye molecules with I^−^ ions favour SS over TZ especially at higher concentrations (125 and 150 ppm) making SS to have higher % IE_WL_ than TZ. However, the reverse is the case at 313 K in which the interactions of TZ molecules with I^−^ ions are more synergistic than SS/I^−^ interactions, which informs higher inhibition potentials of TZ. The % IE_WL_ values of SS, TZ and AM (without KI) at 313 K are relatively close, whereas the values are clearly different in the presence of KI. This again confirms synergistic interactions between the molecules of the dye and I^−^ ions. It has been reported in literature [47] that anions such as CI^−^, I^−^, SO_4_^2−^ and S^2−^ have the ability of taking part as reaction intermediates on corroding metal surface, which can either inhibit or stimulate corrosion reaction. Halides are known to have ability to replace the hydroxyl ions adsorbed on the metal surface, therefore reducing the catalytic effect of the hydroxyl ions [47]. Therefore, the increase in % IE_WL_ with the addition of KI is attributed to the fact that I^−^ ions improve adsorption of the inhibitor molecules on the metal surface [48]. The % IE_WL_ values generally decrease with increase in temperature. Effect of temperature on the inhibited acid- metal reaction is very intricate because many changes may occur on the metal surface at higher temperatures. Possible changes include rapid etching, desorption and/or decomposition of the inhibitor, increase in the solubility of substance that might impede corrosion rate such as the protective film and/or any reaction products precipitated on the metal surface [20,47,48]. The distinctly higher % IE_WL_ of FG compared to the other four dyes is due to the presence of more number of aromatic rings, π-electrons, O and S heteroatoms in its molecules compared to the other dyes (FG > AR > AM > TZ > SS).

#### 2.1.2. Thermodynamic and Activation Parameters

Adsorption is an important process in corrosion inhibition because the inhibition of metal corrosion by organic molecules is often due to adsorption of the inhibitor molecules onto the metal surface thereby blocking the active sites that are susceptible to corrosion reaction. A better understanding of the adsorption behaviour of an inhibitor can be achieved by investigating the thermodynamics of the adsorption process. The dependence of the corrosion rate on temperature can be expressed using the Arrhenius equation:

(1) $$\log C_{R}=\log A-\frac{E_{a}}{2.303RT}$$

where *C_R_* is the corrosion rate (g·cm^−2^·h^−1^), *E_a_* is the apparent activation energy, *R* is the molar gas constant (8.314 JK^−1^·mol^−1^), *T* is the absolute temperature and *A* is the frequency factor. The values of the standard enthalpy and entropy of activation, ΔH* and ΔS* respectively were calculated from the transition state equation:

(2) $$\text{log}\left(C_{R}\mathrm{/T}\right)=\left[\text{log}\left(\frac{R}{Nh}\right)+\left(\frac{∆S*}{\text{2.303}R}\right)\right]\text{+}\left(-\frac{∆H*}{\text{2.303}R}\right)\left(\frac{1}{T}\right)$$

where *h* is Planck’s constant and *N* is the Avogadro number.

The Arrhenius plots (log*C_R_* *vs.* 1/*T*) and the transition state plots (log (*C_R_*/*T*) *vs.* 1/*T*) for mild steel corrosion in 0.5 M HCl without and with various concentrations of SS, AM, AR, TZ, and FG, in the absence and presence of KI are shown in Figures S1 and S2 respectively. Both the Arrhenius and the transition state plots (Figures S1 and S2 respectively) exhibited adequate linearity as they all gave correlation coefficient (R^2^) values of up to 0.9 and above. The values of the apparent activation energy (*E_a_*) were calculated from the slope of the Arrhenius plots (slope = −*E_a_*/2.303*R*), while the values of ΔH* and ΔS* were respectively obtained from the slope (slope = −ΔH*/2.303*R*) and intercept (intercept = log(*R/Nh*) + ΔS*/2.303*R*) of the transition state plots. The values of *E_a_*, ΔH* and ΔS* are listed in Table 1. The results in Table 1 include the thermodynamic parameters for the studied dyes at various concentrations without and with addition of KI. It is obvious from the results that the values of *E_a_* in the absence of KI generally increase with increasing concentration of the inhibitors. An increase in *E_a_* with increase in inhibitor concentration is an indication of physical adsorption process at the initial stage of inhibition [49,50,51,52,53] and it suggests increase in inhibition efficiency with increase in concentration of the inhibitors. Such generalization could not be made in the presence of KI as the values of *E_a_* did not follow a definite pattern as the concentration of the inhibitors increased. The decrease in *E_a_* with increasing concentration observed in some cases, particularly upon addition of KI may be due to a shift of the net corrosion reaction from the uncovered surface to the portions of the surface with the adsorbed inhibitors such that the net corrosion reaction now directly involves the sites with the adsorbed inhibitor molecules [54]. A lower value of *E_a_* in the presence of an inhibitor may also be due to a slow rate of inhibitor adsorption with a resultant closer approach to equilibrium during the experiments at higher temperature [55,56]. A more detailed discussion on various possibilities associated with lower values of *E_a_* in the presence of inhibitors was also provided by Bernali *et al.* [57].

**Table 1:** Activation parameters *E_a_*, ΔH* and ΔS* derived from the Arrhenius plots in the absence and presence of different concentrations of the studied dyes.

Inhibitor	Inhibitor Concentration (ppm)	*E_a_* (kJ·mol^−1^)	ΔH* (kJ·mol^−1^)	ΔS* (JK^−1^·mol^−1^)
	-	58.28	55.26	−115.47
SS	25	77.72 (90.96) ^a^	75.15 (88.15) ^a^	−58.52 (−20.94) ^a^
	50	78.09 (90.94) ^a^	75.46 (88.00) ^a^	−57.61 (−22.02) ^a^
	75	79.28 (91.91) ^a^	77.08 (89.48) ^a^	−52.70 (−17.78) ^a^
	100	81.27 (94.15) ^a^	78.43 (91.46) ^a^	−48.79 (−12.30) ^a^
	125	82.38 (93.77) ^a^	80.03 (91.70) ^a^	−44.06 (−6.39) ^a^
	150	82.76 (95.98) ^a^	80.53 (93.13) ^a^	−42.64 (−8.31) ^a^
AM	25	81.50 (91.88) ^a^	78.83 (89.40) ^a^	−46.55 (−18.62) ^a^
	50	81.33 (92.95) ^a^	78.75 (60.60) ^a^	−47.05 (−15.12) ^a^
	75	84.63 (96.10) ^a^	81.84 (93.17) ^a^	−37.74 (−7.64) ^a^
	100	85.05 (95.89) ^a^	79.78 (93.30) ^a^	−44.22 (−7.56) ^a^
	125	85.99 (95.56) ^a^	83.53 (92.60) ^a^	−32.92 (−9.80) ^a^
	150	87.81 (95.70) ^a^	85.15 (93.21) ^a^	−28.09 (−6.73) ^a^
AR	25	58.28 (96.14) ^a^	91.94 (87.69) ^a^	−9.14 (−7.14) ^a^
	50	81.69 (102.16) ^a^	79.64 (89.46) ^a^	−39.82 (−6.81) ^a^
	75	83.64 (97.44) ^a^	81.99 (89.99) ^a^	−37.49 (−2.57) ^a^
	100	85.20 (98.75) ^a^	82.96 (87.16) ^a^	−34.66 (9.40) ^a^
	125	86.22 (99.58) ^a^	84.59 (91.59) ^a^	−29.84 (9.24) ^a^
	150	87.61 (98.05) ^a^	88.91 (91.20) ^a^	−16.87 (−2.15) ^a^
TZ	25	77.33 (89.47) ^a^	74.73 (87.69) ^a^	−59.19 (−23.69) ^a^
	50	81.86 (85.65) ^a^	107.39 (89.46) ^a^	36.84 (−18.53) ^a^
	75	82.88 (92.85) ^a^	106.97 (89.99) ^a^	34.76 (−17.37) ^a^
	100	83.47 (92.85) ^a^	101.74 (87.16) ^a^	23.12 (−25.77) ^a^
	125	85.42 (94.44) ^a^	82.35 (91.59) ^a^	−36.41 (−13.04) ^a^
	150	85.63 (93.90) ^a^	90.91 (91.20) ^a^	−10.30 (−14.96) ^a^
FG	25	108.63 (59.26) ^a^	106.08 (57.03) ^a^	33.01 (−129.52) ^a^
	50	109.78 (66.77) ^a^	107.39 (64.44) ^a^	36.84 (−107.41) ^a^
	75	109.95 (63.54) ^a^	106.97 (60.95) ^a^	34.76 (−119.96) ^a^
	100	105.78 (69.77) ^a^	105.85 (66.95) ^a^	30.27 (−102.17) ^a^
	125	108.75 (67.78) ^a^	106.07 (64.58) ^a^	30.52 (−109.99) ^a^
	150	106.23 (68.47) ^a^	104.38 (64.14) ^a^	23.87 (−106.33) ^a^

^a^: corresponds to results with the addition of KI.

The values of enthalpy, ΔH* and entropy, ΔS* of activation were calculated from the plots of log *C_R_*/*T* *vs.* 1/*T* (Figure S2). The results in Table 1 shows that the values of ΔH* are positive both in the absence and presence of the inhibitor, which implies the endothermic nature of mild steel dissolution process for both instances, without and with the addition of KI [58,59]. The negative values of ΔS* obtained in many cases indicate that the formation of the activated complex in the rate determining step is an associative process rather than dissociative and suggest a decrease in disorderliness as the reaction proceeds from reactants to activated complex [60]. The positive values of ΔS* obtained in the presence of FG (without KI) and in few other cases suggest that the rate-determining step represents a more disordered arrangement, which may involve the decomposition of some intermediate products of corrosion reaction such as dissociation of adsorbed chloride ions or water molecules from the steel surface in order to allow for adsorption of the inhibitor molecules.

### 2.2. Adsorption Isotherms

**Figure 3:**
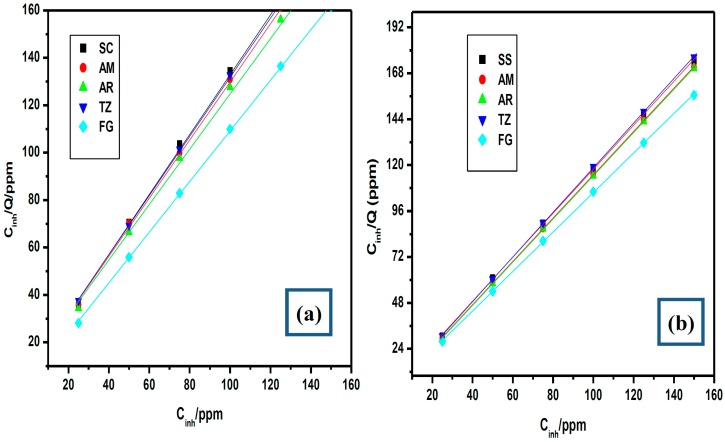
Langmuir adsorption isotherm plots for the adsorption of the studied dyes at 303 K (**a**) without KI and (**b**) with KI.

The adsorption isotherms describe the molecular interactions of the inhibitor molecules with the active sites on the metal surface [61,62,63,64] and provide more insights into the mechanism of corrosion inhibition. To investigate the modes of adsorption of the studied dyes onto mild steel surface, different adsorption isotherms were examined including the Frumkin, Flory-Huggins, Freundlich, Temkin and Langmuir adsorption isotherms. The Langmuir adsorption isotherm was found to provide the best description of the behavior of the investigated food dyes with near unity correlation coefficient (R^2^) value. The adsorption isotherms were plotted for the studied dyes without and with the addition of KI using the linear form of the Langmuir adsorption isotherm:

(3) $$\frac{C_{inh}}{\text{θ}}=\frac{1}{K_{ads}}+C_{inh}$$

where θ is the degree of surface coverage, *K_ads_* is the equilibrium constant of the adsorption/desorption process and *C_inh_* is the concentration of the inhibitor. Only the representative plots at 303 K are shown in Figure 3. Similar observations were made at other temperatures (but not reported here). The change in Gibb’s free energy of adsorption (Δ$G_{ads}^{o}$) was calculated using the equation:

(4) $$\Delta G_{ads}^{o}=-RT\ln\left(55.5K_{ads}\right)$$

where R is the gas constant (8.314 kJ−1·mol−1), 55.5 is the molar concentration (mol·L−1) of water in the solution, *K_ads_* is the equilibrium constant for the adsorption process and *T* is the absolute temperature. The values of *K_ads_* and Δ$G_{ads}^{o}$ were calculated for all the studied dyes without and with addition of KI at various temperatures and the results are presented in Table 2. Generally, the values of Δ$G_{ads}^{o}$ up to −20 kJ·mol^−1^ suggest electrostatic interactions between the charged molecules and the charged metal surface (*i.e.*, physisorption), while those around −40 kJ·mol^−1^ or more negative involve charge sharing or transfer from organic molecules to the metal surface to form coordinate bond (chemisorption) [4,63,64].

The obtained values of Δ$G_{ads}^{o}$ from Table 2 are negative suggesting the spontaneity of the adsorption process. The results also show that the calculated values of Δ$G_{ads}^{o}$ range between −0.93 to −8.49 kJ·mol−1 without KI, and −2.81 to −13.77 with KI, which imply that the adsorption of the studied dyes on mild steel surface in 0.5 M HCl occurred via physisorption mechanism. There is no general trend for the variation of the values of *K_ads_* and Δ$G_{ads}^{o}$ with change in temperature. However, the values of *K_ads_* and Δ$G_{ads}^{o}$ in the presence of KI are indicative of better adsorption in most cases.

**Table 2:** Thermodynamic parameters for adsorption of the studied dyes on mild steel surface at different temperatures.

Inhibitor	T/K	K_ads_ (10^3^ × mol^−1^)	−Δ$G_{ads}^{o}$ (kJ·mol^−1^)
SS	303	0.15 (0.34) ^a^	−5.40 (−7.44) ^a^
	313	0.09 (0.10) ^a^	−4.40 (−4.39) ^a^
	323	0.03 (0.08) ^a^	−1.37 (−3.94) ^a^
	333	0.05 (0.05) ^a^	−2.81 (−2.81) ^a^
AM	303	0.14 (0.55) ^a^	−5.13 (−8.60) ^a^
	313	0.09 (0.17) ^a^	−4.43 (−5.89) ^a^
	323	0.03 (0.12) ^a^	−1.64 (−5.02) ^a^
	333	0.07 (0.07) ^a^	−3.79 (−3.79) ^a^
AR	303	0.12 (0.63) ^a^	−4.87 (−8.95) ^a^
	313	0.06 (0.28) ^a^	−3.07 (−7.18) ^a^
	323	0.03 (0.12) ^a^	−0.93 (−5.11) ^a^
	333	0.11 (0.11) ^a^	−4.86 (−4.86) ^a^
TZ	303	0.14 (0.49) ^a^	−5.21 (−8.32) ^a^
	313	0.097 (0.23) ^a^	−4.39 (−6.59) ^a^
	323	0.03 (0.09) ^a^	−0.93 (−4.49) ^a^
	333	0.10 (0.10) ^a^	−4.71 (−4.61) ^a^
FG	303	0.40 (0.39) ^a^	−8.04 (−7.76) ^a^
	313	0.47 (3.57) ^a^	−8.49 (−13.77) ^a^
	323	0.01 (0.41) ^a^	−1.17 (−8.37) ^a^
	333	0.01 (0.50) ^a^	−1.69 (−8.89) ^a^

^a^: corresponds to results with the addition of KI.

### 2.3. Electrochemical Measurements

#### Potentiodynamic Polarization Measurements

**Figure 4:**
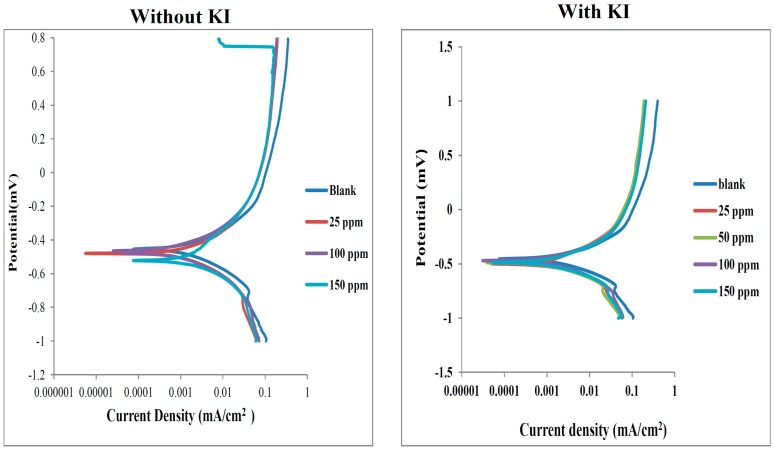
Potentiodynamic polarization curves for mild corrosion in 0.5 M HCl without and with various concentrations of SS without KI (**left-hand side**) and with KI (**right-hand side**).

Potentiodynamic polarization measurements were carried out in order to study the electrochemical kinetics of the reactions. The potentiodynamic polarization curves for mild steel in 0.5 M HCl without and with various concentrations of SS and in the absence and presence of KI are presented in Figure 4. The potentiodynamic polarization curves for mild steel in 0.5 M HCl without and with various concentrations of the other four dyes and in the absence and presence of KI showed similar behaviour and are presented in Figure S3. The polarization curves show similar features in the absence and presence of the inhibitors and also without and with addition of KI. This suggests that the inhibitors (without or with KI) retard mild steel corrosion only by blocking the active site on the steel, without changing the mechanism of corrosion reaction [65]. Electrochemical kinetic parameters such as the corrosion current density (*i_corr_*), anodic (*b_a_*) and cathodic (*b_c_*) Tafel slopes were obtained from the polarization curves by extrapolating the Tafel regions of the curves to the corrosion potential (*E_corr_*). The percentage inhibition efficiency (% *IE_P_*) values were calculated from the *i_corr_* values using the equation:

(5) $$\%IE_{P}=\frac{i_{corr}^{0}-i_{corr}^{i}}{i_{corr}^{0}}\times 100$$

where $i_{corr}^{0}$ and $i_{corr}^{i}$ are values of corrosion current density in the absence and presence of inhibitor respectively. The results of the electrochemical parameters and the % *IE_P_* are listed in Table 3. An inhibitor can be regarded as anodic or cathodic type when the shift in *E_corr_* in the presence of the inhibitor when compared to the blank is less than 85 mV [4,6,65,66]. In the present study, the shift in *E_corr_* is less than 85 mV both without and with addition of KI. This implies that the studied dyes are mixed-type inhibitors both in the absence and presence of I^−^ ions. That is, the studied dyes inhibit the anodic dissolution of mild steel in the acid as well as the cathodic half-reaction involving hydrogen ion reduction. The results in Table 3 also reveal that the values of b_a_ and b_c_ in the presence of the inhibitors are generally lower than the values for the blank acid system, which suggests that the inhibitors reduce the rate of mild steel corrosion by retarding both the anodic and cathodic reactions. The values of the Tafel slopes in the presence of the inhibitors appear to decrease with increasing concentration of the inhibitors particularly without the KI addition. The change in Tafel slopes with change in concentration has been attributed to some factors such as the composition of the working electrode, concentration of the electrolyte, scan rate, and charge transfer coefficient [67]. In a particular study [68], the change in anodic Tafel slope with varying concentration of inhibitor was attributed to the redox formation of Fe(II) and Fe(III) complexes of the inhibitor, which was affected by the pH of the medium and concentration of the inhibitor. The highest value of % IE was obtained for each inhibitor upon addition of KI. Though, the trends of % IE both in the absence and presence of KI cannot be generalized. No specific reason could be provided for this non-uniform trend, but one possible explanation is the complex nature of the electrochemical system that features prospective aggregation of dye molecules as well as interactions with I^−^ ions which can be affected by the concentration of the dyes.

**Table 3:** Potentiodynamic polarization parameters such as corrosion rate, corrosion current density (*i_corr_*), corrosion potential (*E_corr_*), and anodic and cathodic Tafel slopes (*b_a_* and *b_c_*) and corrosion rate using different dye inhibitors with and without KI.

Inhibitor/Blank	Conc. of Inhibitor (ppm)	*i_corr_* (A cm^−2^) × 10^−4^	*b_a_* (mV·dec^−1^)	*b_c_* (mV·dec^−1^)	−*E_corr_* (mV)	*% IE_P_*
Blank	-	16.86	148	147	459	
SS	25	8.190 (7.275) ^a^	111 (80) ^a^	101 (150) ^a^	481 (505) ^a^	51.42 (56.85) ^a^
	100	5.588 (6.459) ^a^	93 (78) ^a^	100 (76) ^a^	478 (490) ^a^	66.86 (61.69) ^a^
	150	4.613 (4.720) ^a^	89 (76) ^a^	75 (89) ^a^	477 (488) ^a^	72.94 (72.04) ^a^
AM	25	5.982 (3.846) ^a^	100 (67) ^a^	93 (122) ^a^	437 (490) ^a^	64.52 (77.19) ^a^
	50	5.122 (3.463) ^a^	82 (81) ^a^	93 (133) ^a^	458 (491) ^a^	72.16 (79.46) ^a^
	150	4.489 (5.078) ^a^	79 (87) ^a^	81 (103) ^a^	460 (481) ^a^	73.37 (69.88) ^a^
AR	25	7.797 (1.659) ^a^	122 (168) ^a^	109 (189) ^a^	463 (475) ^a^	53.75 (90.16) ^a^
	100	7.425 (5.086) ^a^	57 (97) ^a^	96 (109) ^a^	524 (460) ^a^	55.96 (69.83) ^a^
	150	7.272 (4.598) ^a^	76 (80) ^a^	95 (89) ^a^	483 (483) ^a^	56.86 (72.73) ^a^
TZ	25	4.504 (1.388) ^a^	112 (154) ^a^	125 (151) ^a^	461 (465) ^a^	73.29 (91.77) ^a^
	100	3.554 (4.422) ^a^	72 (79) ^a^	100 (91) ^a^	481 (476) ^a^	78.92 (73.77) ^a^
	150	2.902 (7.256) ^a^	49 (91) ^a^	61 (122) ^a^	510 (489) ^a^	82.79 (56.96) ^a^
FG	25	9.327 (7.333) ^a^	102 (101) ^a^	144 (137) ^a^	486 (485) ^a^	44. 76 (56.51) ^a^
	100	5.800 (3.953) ^a^	90 (64) ^a^	112 (116) ^a^	473 (482) ^a^	65.59 (76.55) ^a^
	150	5.152 (1.763) ^a^	69 (36) ^a^	121 (80) ^a^	490 (480) ^a^	69.44 (89.53) ^a^

^a^: corresponds to results with the addition of KI.

### 2.4. Synergism Consideration

The synergism parameter, *S_I_*, was evaluated using the relationship given by Aramaki and Hackerman and reported elsewhere [23,48]:

(6) $$S_{I}=\frac{1-I_{1+2}}{\text{I}-I_{1+2}^{\prime }}$$

where *I*_1+2_ = *I*_1_ + *I*_2_; *I*_1_ = inhibition efficiency of the iodide ions; *I*_2_ = inhibition efficiency of the inhibitor and *I′* = measured inhibition efficiency for inhibitor in combination with I^−^ ions. This parameter was evaluated from the inhibition efficiency values obtained from both the weight loss and potentiodynamic polarization measurements. The results obtained are presented in Table 4 for different concentrations of the inhibitors and are found to be greater than unity. This indicates that the improved inhibition efficiency caused by the addition of I^−^ ions to the inhibitors is only due to synergistic effect. Similar results have been reported in literatures [28,47]. The synergistic interactions suggested by the values of S_I_ can be assumed to occur via initial chemisorption of the I^−^ ions on the steel surface followed by physisorption of the protonated inhibitor molecules. That is, the electrostatic interactions between the protonated form of the inhibitors and the metal surface may be facilitated by the already chemisorbed I^−^ ions, which in this case assumed the role of interlayer species. Stabilization of the adsorbed I^−^ ions with cationic inhibitors may lead to a greater surface coverage and therefore greater inhibition potential. It could therefore be assumed that the addition of I^−^ ions enhances the inhibition efficiency to a considerable extent due to increase in the surface coverage in the presence of I^−^ ions.

**Table 4:** Synergistic parameters (S_I_).

Inhibitor	S_I_ Values at Various Concentrations of Inhibitor					
	25 ppm	50 ppm	75 ppm	100 ppm	125 ppm	150 ppm
SS	1.50 (1.83) ^b^	1.50	1.49	1.50 (1.94) ^b^	1.52	1.50 (1.74) ^b^
AM	1.45 (1.52) ^b^	1.45	1.48	1.48 (1.00) ^b^	1.49	1.50 (1.80) ^b^
AR	1.47 (1.18) ^b^	1.49	1.50	1.50 (1.55) ^b^	1.52	1.55 (1.50) ^b^
TZ	1.46 (1.37) ^b^	1.51	1.52	1.52 (1.78) ^b^	1.53	1.53 (2.39) ^b^
FG	1.57 (1.72) ^b^	1.54	1.53	1.52 (1.54) ^b^	1.52	1.52 (1.36) ^b^

^b^: = electrochemical results.

### 2.5. Quantum Chemical Calculation

#### 2.5.1. Results of the Study on Neutral Species

**Figure 5:**
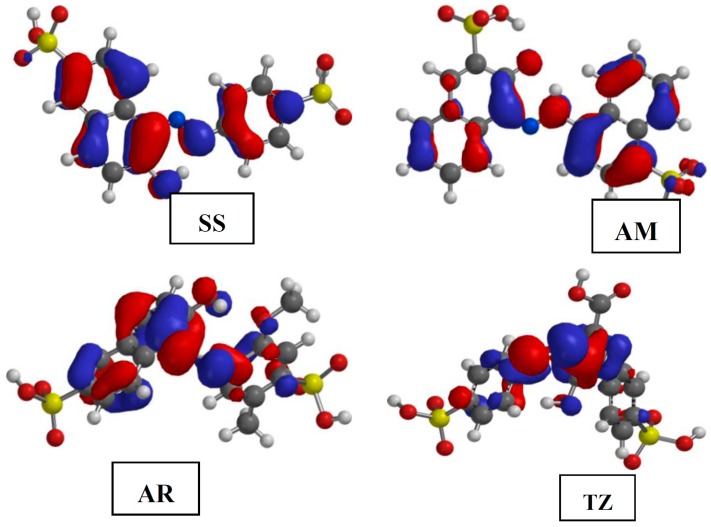
The highest occupied molecular orbitals (HOMO) electron density surfaces for the studied dyes.

**Figure 6:**
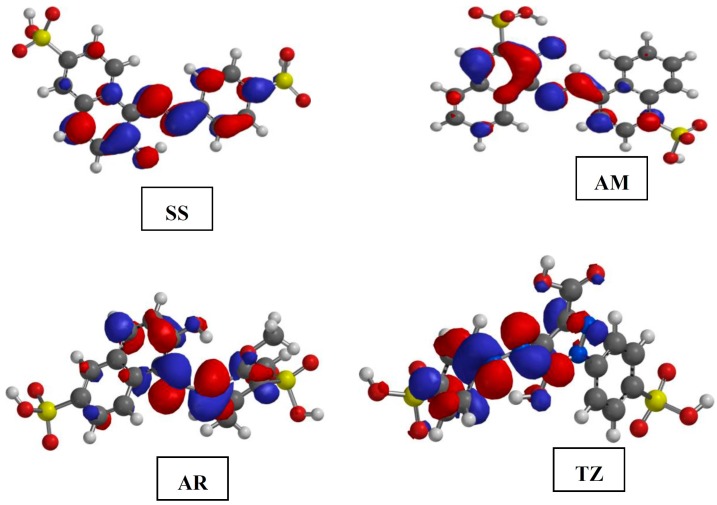
The lowest unoccupied molecular orbitals (LUMO) electron density surfaces for the studied dyes.

The gas phase optimized geometries of the studied dyes are shown in Figure S4. The optimized geometries were confirmed to correspond to energy minima by the absence of imaginary frequencies in the vibrational frequency calculations. All quantum chemical parameters used in comparison with experimental results are those derived from the ground state optimized geometries of the studied compounds. The frontier molecular orbitals (FMO) provide information about the reactive sites of the inhibitors and the nature of orbitals available in an inhibitor molecule. The highest occupied molecular orbitals (HOMO) and lowest unoccupied molecular orbitals (LUMO) of the inhibitors are often used to describe prospective donation and retro-donation mechanism between the inhibitor molecules and metal atom. The HOMO and LUMO electron density surfaces of the studied dyes are shown in Figure 5 and Figure 6, respectively. The numbering patterns of the atoms in each molecule as shown in Figure 1 are adopted for the discussion of the results of the quantum chemical studies.

The HOMO provides information about the sites of the inhibitor molecules that are most likely to donate electrons to the appropriate vacant orbitals of the metal atom to afford interactions that will lead to adsorption and corrosion inhibition. The HOMO of SS comprises both σ- and π-type orbitals and it is largely distributed over the C atoms of the aromatic rings, the azo-N atom and the O-atom of the hydroxyl group. The –SO_3_H groups are not involved in the HOMO. This implies that the SS molecule has the tendency of donating its HOMO σ- or π-electrons to the appropriate vacant p-orbitals or d-orbitals of the metal atom. The HOMO of AM also comprises both σ- and π-type orbitals but essentially π-type. The π-type HOMO is distributed over the C atoms of the naphthalene rings, while the σ-type HOMO is mainly concentrated on the –OH group and one of the azo N-atoms. This suggests that the AM is capable of donating π-electrons from the aromatic naphthalene rings to the empty d-orbitals of metal atom. Under favourable conditions, the AM molecule can also interact with the metal atom via donation of the HOMO σ-electrons from its –OH or azo N-atom to the appropriate vacant p-orbital of the metal atom. In the AR molecule, the HOMO is also distributed largely on the C=C double bonds of the naphthalene ring, and also extended to the N-atom of the azo and O-atom of the hydroxyl groups. The HOMO of TZ comprises both σ- and π-orbitals being essentially distributed over the C and N atoms of the pyrazole ring, the azo –N=N– group, and some C atoms on the phenyl groups. The TZ molecule has a better chance of donating π-electrons from the pyrazole ring to the appropriate vacant d-orbitals of metal atom to form coordinate bond.

The LUMO provides information about the regions in a molecule that possess the highest tendency to accept electrons from an electron rich species. For the purpose of inhibitor-metal interactions, the LUMO of the inhibitor molecule is a pointer to the sites of the molecule at which electrons are most likely to be received from the appropriate occupied orbitals of the metal atom during retro-donation. As shown in Figure 6, the LUMO of SS is mainly distributed on C13=C12, C11, N16, N17≡C18, C19, C20, C23 and C21. In AM, the LUMO is mostly distributed on C5=C6, C6=C7, C7=C8, C10=C9 of the naphthalene ring attached at N16 and also extended to N16, N17≡C18, C19 and C21. The LUMO of AR is mainly distributed on C8, C5, C9, C10 on the benzene ring attached at N14, and also on N15, C17=C18, C19 and C21. In TZ, the electron density of the LUMO spreads over the C5, C9, C6=C7, and C8≡N11 on the benzene ring attached at N11. These regions suggest the most probable sites of electron-acceptance in the studied dye molecules when electrons are being back-donated from the appropriate occupied orbitals of the metal atom.

The energy values of the HOMO and LUMO, *i.e.*, E_HOMO_ and E_LUMO_ respectively provide further information about reactivity of a molecule. The higher the E_HOMO_ the better the ability of a molecule to donate electrons to an electron poor specie, while the lower the E_LUMO_ the better the chance of a molecule to accept electron from electron rich specie. The values of the E_HOMO_ and E_LUMO_ of the studies compounds are listed in Table 5. The results reveal that the order of increasing E_HOMO_ is TZ < SS < AM < AR. This indicates that TZ has least tendency to donate electrons to the metal surfaces, while AR has the highest tendency to donate electrons to the appropriate vacant orbitals of the metal atom. This is good correlation between the trend of the E_HOMO_ and the trend of the highest value of experimentally determined % IE as shown in Table 5. The trend of the E_LUMO_ for the studied dyes is AR < TZ < SS < AM, which suggests that AR has the least tendency to accept electrons from metal atom while AM has the highest tendency to accept electrons from the suitable occupied orbitals of the metal during back-donation. The trend of the E_LUMO_ does not agree with the experimental % IE. The energy gap, ΔE (ΔE = E_LUMO_ − E_HOMO_) also provides important information about the reactivity of a molecule, the smaller the ΔE for an inhibitor molecule, the greater the reactivity of the molecule, which suggests a better chance of interaction with metal surface. The values of ΔE in Table 5 suggest that AM has the lowest value of ΔE, while TZ has the highest value.

**Table 5:** Selected quantum chemical parameters for the studied dyes considering both the neutral and the protonated species.

Parameters	AR		AM		SS		TZ	
	Neutral	Protonated	Neutral	Protonated	Neutral	Protonated	Neutral	Protonated
E_HOMO_ (eV)	−6.15	−9.59	−6.35	−9.29	−6.46	−9.96	−6.62	−9.05
E_LUMO_ (eV)	−2.62	−7.09	−3.63	−7.12	−3.27	−7.46	−2.87	−7.55
ΔE (eV)	3.53	2.5	2.72	2.17	3.19	2.5	3.75	1.5
η (eV)	1.77	1.25	1.36	1.085	1.60	1.25	1.88	0.75
σ (eV)	0.57	0.8	0.74	0.92	0.63	0.8	0.53	1.33
ΔN	−0.74	−0.54	−0.74	−0.56	−0.67	−0.68	−0.60	−0.87
ω	5.45	27.82	9.15	31.02	7.42	30.35	6.00	45.93
μ (Debye)	6.14	5.65	6.73	8.53	3.74	8.11	5.28	12.40
IE (%)	87.94		87.65		86.03		85.06	

Other quantum chemical parameters including the global hardness (η), global softness (σ), fraction of electrons transferred (ΔN) from the inhibitor to the metal atom, global electrophilicity (ω) and dipole moment (μ) were also calculated and listed in Table 5. Apart from ΔN, whose magnitude is in the order AR = AM > SS > TZ, which agrees with the trend of % IE listed in Table 5, all other quantum chemical parameters do not provide direct correlation with the experimental % IE. The disagreement between individual quantum chemical parameters and experimental % IE is not an unknown conjecture. An attempt to correlate individual quantum chemical parameters to the experimental inhibition efficiencies of the inhibitors does not always reveal optimal correlation [27]. Such disagreement has been reported in literature and it is often attributed to the complex nature of adsorption phenomenon that might exist between the inhibitor molecules and metal atom. In such instances, it is often useful to adopt the quantitative structure activity relationship (QSAR) approach in which two or more quantum chemical parameters are brought together to form a composite index which can then be used to correlate experimental inhibition efficiencies [69].

The Mulliken atomic charges were calculated for each atom in the studied inhibitor molecules and the results are displayed in Figure S5. The Mulliken atomic charges show the amount of charges condensed on each atom in the molecule and suggests the atom in the molecule that is most susceptible to electrophilic or nucleophilic attack. Atoms with highest negative charge in the molecule are often susceptible to electrophilic attack, while those with high positive charges are more prone to nucleophilic attack. The set of atoms with high positive charges in SS are the S atoms of the sulphonic groups, which implies that these atoms have a greater tendency to interact with metal surface if electron attraction is the dominant mechanism. On the other hand, all the O atoms in the SS molecule that possess high negative charges would therefore be the best sites of interaction with positively charged metal surface. The N atoms of the azo group also carry significant amount of negative charges and might also get involved in the interactions with positively charged metal surface. Similar results were obtained for the other dye molecules considered in this study. All the C atoms of the aromatic rings carry intermediate amounts of positive or negative charges.

The Fukui functions are important local reactivity indices often used to predict the prospective centres for nucleophilic and electrophilic attacks in a molecule. The higher the value of the nucleophilic Fukui index, *f*^+^ condensed at a particular atom, the better the chance of the atom to undergo nucleophilic attack. On the other hand, the higher the value of the electrophilic Fukui index, *f*^−^ at an atomic site, the higher the tendency of electrophilic attack at the centre. The values of the *f*^+^ and *f*^−^ obtained for the studied dyes are listed in Table S1. The preferred site for nucleophilic attacks as suggested by the values of *f*^+^ are N16 and N17 in SS, O11 and N16 in AM, C17 and N14 in AR, and N11 and N13 in TZ. On the other hand, the most susceptible sites for electrophilic attacks as inferred from the results of *f*^−^ in Table S1 are C14 and O15 in SS, C7 and C21 in AM, N14 and N15 in AR, and C8 and N11 in TZ. Though it is difficult to make a simple generalization in correlating the magnitudes of the Fukui indices and experimental % IE for different inhibitors, the results in Table S1 revealed that if the adsorption of the studied dyes and hence corrosion inhibition occurred via the interactions of the active atomic sites of the dyes with negatively charged steel surface as usually expected for iron in acidic medium, the higher magnitudes of *f^+^* at C17 and N14 of AR compared to the active centres for nucleophilic attacks in the other dye molecules agree with its higher % IE (as listed in Table 5).

#### 2.5.2. Results of the Study on Protonated Species

**Figure 7:**
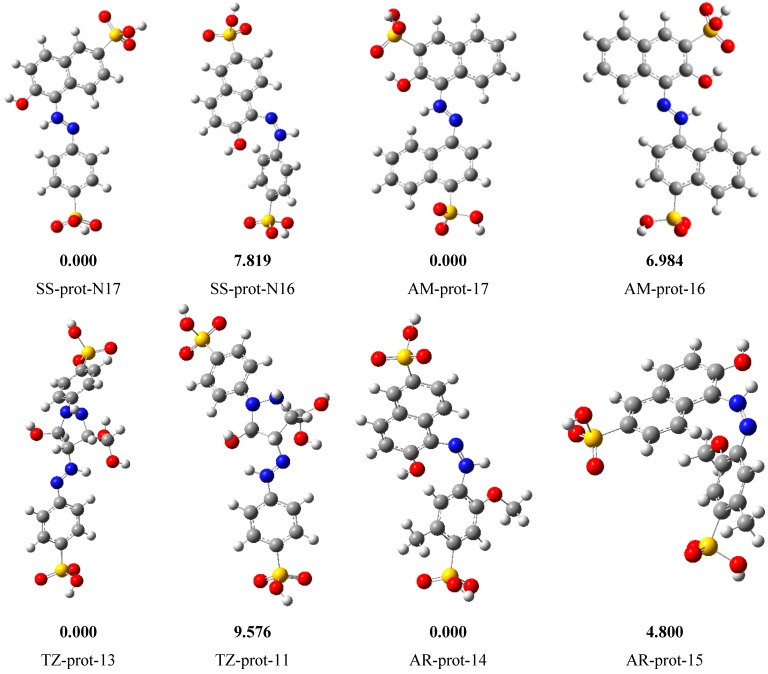
N-protonated species of the studied dyes. The number at the end of the name of the protonated species indicate the site on the molecule at which protonation is considered. Colour legend: White = Hydrogen; Grey = Carbon; Blue = Nitrogen; Red = Oxygen; Yellow = Sulphur.

The presence of heteroatoms in the molecules of SS, AM, AR, and TZ suggests that in acidic solution these compounds may undergo protonation. Therefore, it is important to investigate the protonated forms of the studied structures in order to determine the preferred form of the dyes in acidic solution. The most probable protonation sites (in the considered structures) are the N atoms of the azine group. Since the molecules are not symmetric about the N=N symmetry, it is important to consider the protonation on each N atom in order to determine the preferred site for protonation. The optimized protonated species are shown in Figure 7 together with the corresponding relative energies. As relative energies listed in Figure 7 reveal that the most stable protonated species corresponds to the resulting species due to protonation at N17 in SS, N17 in AM, N13 in TZ and N14 in AR. In all the studied dye molecules, the preferred site for protonation corresponds to a site that results in the formation of the intramolecular hydrogen bond with the neighbouring O atom. The lowest-energy protonated species for each structure has been utilized to obtain information on the reactivity of the protonated species. For the TZ in which both protonated species form intramolecular hydrogen bonds, the lower-energy species, TZ-prot-13 was utilized. Table 5 shows the quantum chemical parameters for the protonated species.

A comparison of the molecular properties between the neutral and the protonated species for the individual structures shows that the protonated form has lower E_HOMO_ than the neutral species which suggests that protonation decreases the tendency of an inhibitor to donate electrons. This phenomenon may be explained as follows: the protonation of a molecule results in an increased nuclear charge so that the nuclear charge pulls more strongly on the outer electrons. As a result, the ionization energy (*i.e.*, the energy required to remove an electron) is higher in the protonated species than in the neutral species. Since the energy of the HOMO is related to the ionization energy (IE) through the equation E_HOMO_ = −IE, an increase in the ionization energy implies a lower E_HOMO_ value. The protonated species also has the lower value of the E_LUMO_ than the neutral species suggesting that protonation increases the tendency of an inhibitor to accept electrons. The protonated species also has the smaller ΔE value, indicating that for each structure the protonated species have higher reactivity than the neutral species. The protonated form has the smaller hardness and lager softness values than the neutral species. However, since the protonated species are less likely to donate electrons, they would rarely be involved in a chemisorption process, which means that the interaction between the protonated species and the metal is through physisoption in which the protonated inhibitors are electrostatically attracted to the metal surface by the already adsorbed Cl^−^ ions [27]. This observation is also in agreement with the physical adsorption mechanism deduced from the experimental results.

## 3. Experimental Section

### 3.1. Materials and Aggressive Solutions

The chemical composition of the mild steel is (wt %): C, 0.17; Mn, 0.46; Si, 0.26; S. 0.017; P, 0.019; and balance Fe. Prior to corrosion test, mild steel was mechanically ground with 200, 400, 600, 800, 1000, and 1200 series of emery papers, washed in acetone and double-distilled water, then dried, and used immediately.

The aggressive solution of 0.5 M HCl was prepared by diluting 37% HCl (E. Merck, Darmstadt, Germany) with double distilled water. The aggressive solutions containing various concentrations (50–250 ppm) of the dyes (used as corrosion inhibitors) were prepared in 0.5 M HCl. All the studied natural dyes were purchased from Merck Chemicals (Darmstadt, Germany) and used without further purification.

### 3.2. Weight Loss Measurements

Weight loss experiments were done according to the method described previously [70]. Weight loss measurements were performed at 308 K (except for temperature effect) for 3 h (except for immersion time effect) by immersing the mild steel coupons into acid solution (100 mL) without and with various concentrations of inhibitors. After the elapsed time, the specimens were taken out, washed, dried, and weighed accurately.

The inhibition efficiency (*E_WL_*, %) and surface coverage (θ) were calculated using the equations:

(7) $$E_{WL}\%=100\left(\frac{w_{0}-w_{i}}{w_{0}}\right)$$

(8) $$\text{θ}=\frac{w_{0}-w_{i}}{w_{0}}$$

where *w*_0_ and *w_i_* are the weight loss value in the absence and presence of inhibitor respectively.

### 3.3. Electrochemical Measurements

A three-electrode cell, consisting of mild steel as working electrode (WE), a platinum rod as counter electrode (CE), and saturated calomel electrode (SCE) as a reference electrode, was used for all electrochemical measurements. All experiments were performed in atmospheric condition without stirring.

The electrochemical impedance spectroscopy (EIS) measurements were carried out in a frequency range from 100 to 0.00001 kHz under potentiostatic conditions at 10 mV peak-to peak amplitude, using the AC signal at corrosion potential (*E_corr_*). The potentiodynamic polarization curves were recorded in the potential range of −250 to +250 mV at a scan rate of 1 mV·s^−1^. The linear polarization resistance (LPR) study was carried out by sweeping the potentials from the cathodic to the anodic potentials within −20 to + 20 mV (*vs*. OCP) at a scan rate of 0.125 mV·s^−1^. All potentials were measured against SCE.

### 3.4. Quantum Chemical Calculations

Quantum chemical calculations were performed using the density function theory (DFT) method comprising the Becke’s three parameter hybrid functional together with the Lee-Yang-Parr correlation functional (B3LYP) [71]. The 6-31G(d,p) basis set was used for all the calculations. All the calculations were performed without symmetry constraint using the Gaussian 03, Version E.01 [72]. It is noteworthy that quantum chemical parameters were reported for only SS, AM, AR and TZ as our available computational resources as at the time of writing this report were not adequately fast enough to optimize the FG molecule. In addition, the molecular structure of FG differs clearly from the other four dyes and as a result grouping its quantum chemical parameters with the other four dyes may not provide an optimum correlation with experimental results. The calculated parameters include energy of the highest occupied molecular orbital (E_HOMO_), energy of the lowest unoccupied molecular orbital (E_LUMO_), energy gap (ΔE), dipole moment (μ), global softness (σ), global hardness (η), electrophilicity (ω), fraction of electrons transferred (ΔN), and electronegativity (χ).

Electronegativity (χ) is the measure of the power of an electron or group of atoms to attract electrons towards itself and it can be estimated by using the equation:

χ = −½ (E_HOMO_ + E_LUMO_) (9)

Global hardness (η) measures the resistance of an atom to a charge transfer and was estimated using the equation:

η = −½ (E_HOMO_ − E_LUMO_) (10)

Global electrophilicity index (ω) was estimated by using the electronegativity and chemical hardness parameters through the equation:

ω = χ^2^/2η (11)

A high value of electrophilicity describes a good electrophile while a small value of elecrophilicity describes a good nucleophile.

Global softness (σ), describes the capacity of an atom or group of atoms to receive electrons, and it was estimated by using the equation:

σ = 1/η = −2/(E_HOMO_ − E_LUMO_) (12)

The fraction of electrons transferred (Δ*N*) [69] from the inhibitor (donating) molecule to the metallic (Fe) atom (acceptor) was calculated using the equation:

(13) $$\Delta N=\frac{{\text{χ}}_{Fe}-{\text{χ}}_{inh}}{2\left(\eta_{Fe}+\eta_{inh}\right)}$$

where χ*_Fe_* and η*_inh_* denote the electronegativity and hardness of iron and inhibitor respectively. A value of 7 eV/mol was used for the χ*_Fe_*, while η*_Fe_* was equated to 0 eV/mol for bulk Fe atom.

The Fukui functions (*f* (**r**)) have been successfully used to analyze the active atomic sites of inhibitor molecules [73,74]. The Fukui functions (*f* (**r**)) indicate the change in the electron density of an *N* electron system upon addition (*f*^+^ (**r**)) or removal (*f*^−^ (**r**)) of an electron [75]. Atom condensed Fukui functions using the Mulliken population analysis (MPA) and the finite difference (FD) approximations approach introduced by Yang and Mortier [76] were calculated using the equations:

(14) $$f_{k}^{+}={\text{ρ}}_{k(N+1)}(r)-{\text{ρ}}_{k(N)}(r)$$

(15) $$f_{k}^{-}={\text{ρ}}_{k(N)}(r)-{\text{ρ}}_{k(N-1)}(r)$$

where ρ*_k_*_(*N*+1)_, ρ*_k_*_(*N*)_ and ρ*_k_*_(*N*−1)_ are the electron densities of the (*N* + 1)-, (*N*)*-* and (*N* − 1)-electron systems respectively and approximated by Mulliken gross charges; $f_{k}^{+}$ and $f_{k}^{-}$ are the Fukui indices condensed on atom *k* and measure its electrophilic and nucleophilic tendencies respectively.

## 4. Conclusions

The corrosion inhibition properties of some organic food dyes, namely SS, AM, AR, TZ and FG on mild steel in 0.5 M HCl, have been investigated using weight loss, potentiodynamic polarization and quantum chemical calculations methods. The synergistic effects of I^−^ ions on the inhibition potentials of these dyes were also reported. The following conclusions can be drawn from the results:

(a) All the studied dyes showed appreciable inhibition efficiency for mild steel corrosion in 0.5 M HCl and their inhibition efficiencies increase with increasing concentration, and decrease with increasing temperature.
(b) The results of weight loss measurements suggest possible aggregates formation of the dyes molecules at some concentrations and temperatures leading to a non-uniform trend of inhibition efficiency.
(c) Addition of KI synergistically improved the inhibition efficiency of all the studied dyes.
(d) Potentiodynamic polarization results revealed that the studied dyes are mixed-type inhibitors both in the absence and presence of KI.
(e) The adsorption of the studied dyes on mild steel surface with and without KI obeys the Langmuir adsorption isotherm and involves physical adsorption mechanism.
(f) The quantum chemically derived E_HOMO_ results give a good correlation with the trend of the inhibition efficiencies of the dyes at 150 ppm and 303 K as obtained from the weight loss measurements. The HOMO, LUMO and Fukui indices revealed the most probable sites of adsorption of the dyes to the steel surface.

## Supplementary Materials

Supplementary materials can be accessed at: http://www.mdpi.com/1420-3049/20/09/16004/s1.
