# Supplementary material for: Adsorption and Corrosion Inhibition Studies of Some Selected Dyes as Corrosion Inhibitors for Mild Steel in Acidic Medium: Gravimetric, Electrochemical, Quantum Chemical Studies and Synergistic Effect with Iodide Ions
Source: Molecules. 2015 Sep 2;20(9):16004–29. doi: 10.3390/molecules200916004 (PMC6331870; doi:10.3390/molecules200916004)
Supplement: Supplementary file 1 [file molecules-20-16004-s001.pdf]

Supplementary Materials

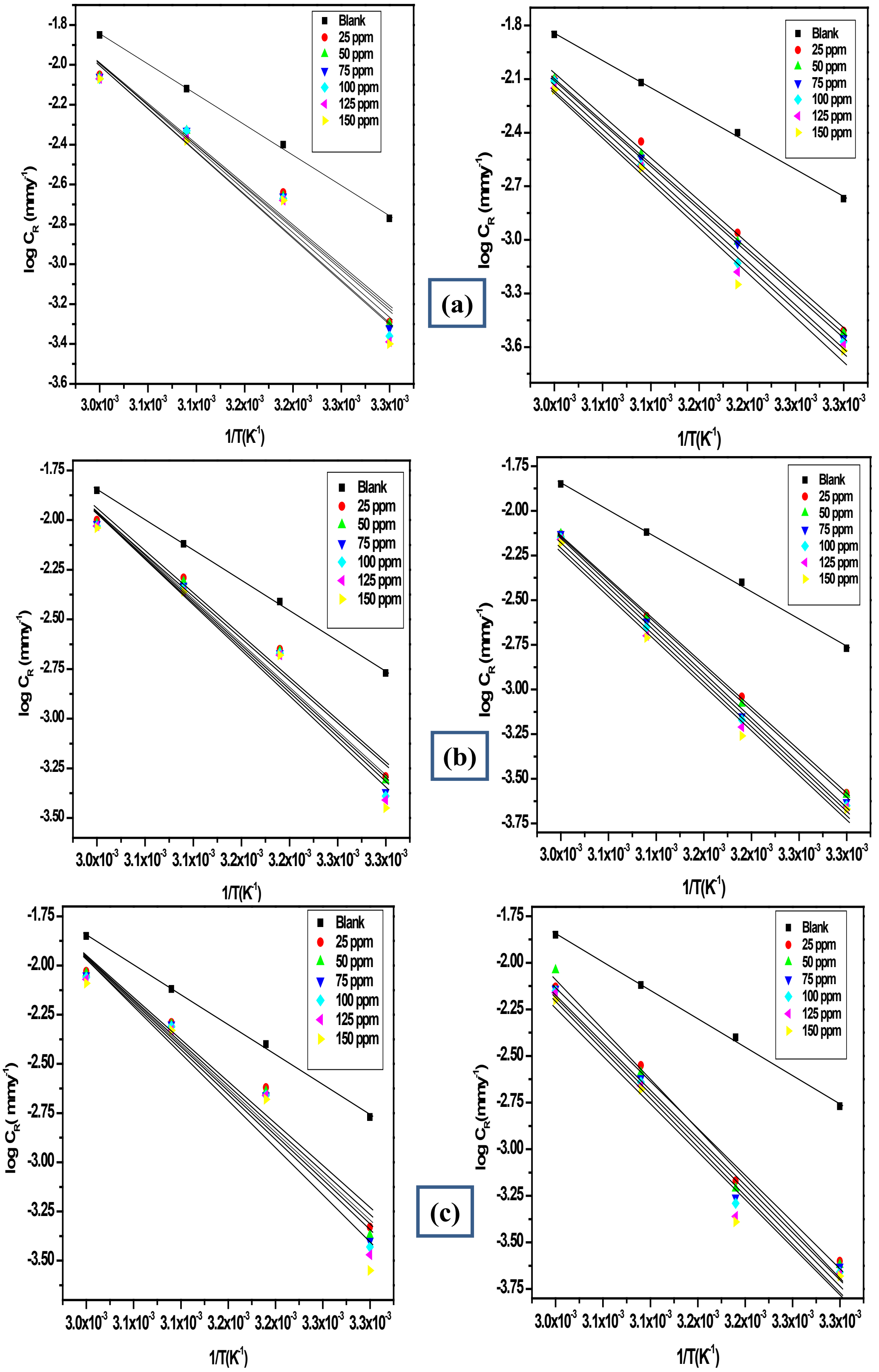

Figure S1. *Cont.*

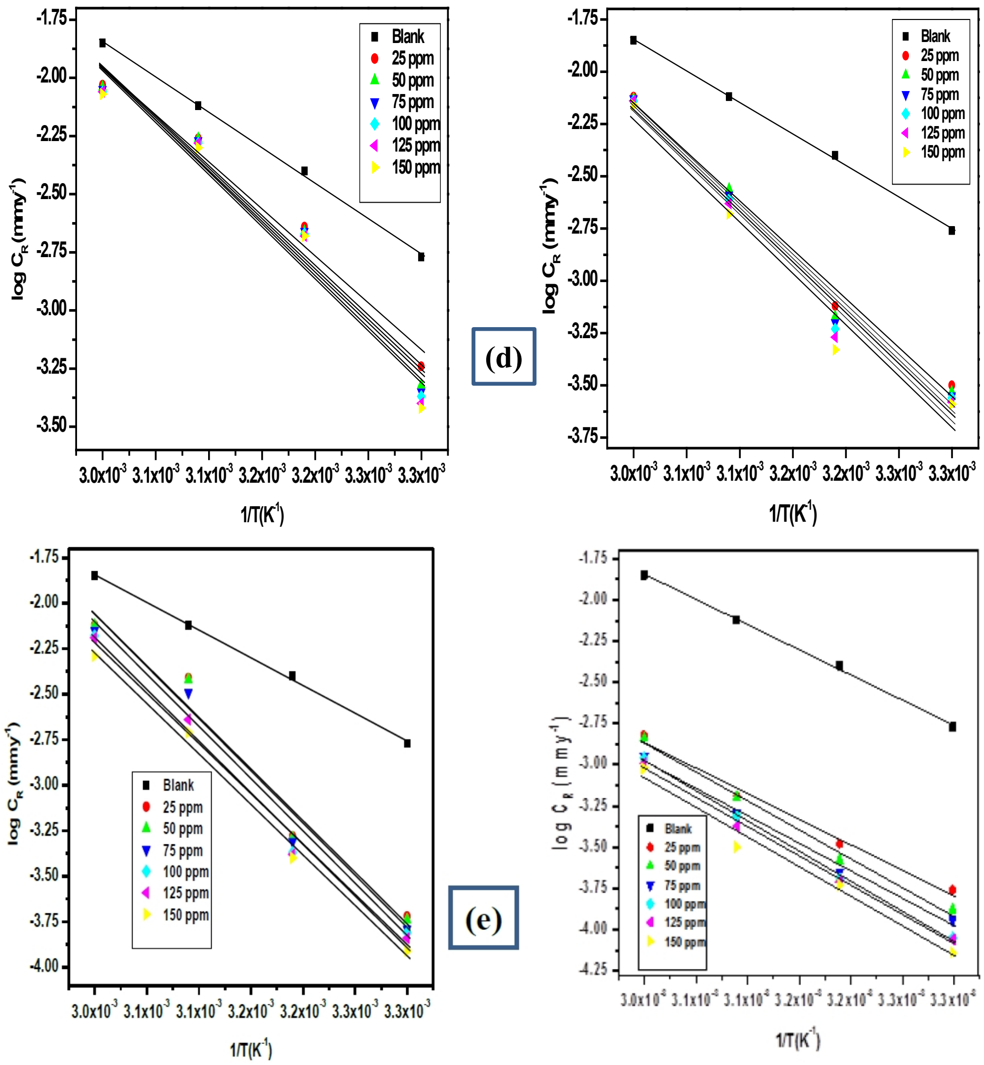

Figure S1. Arrhenius plots (log CR *vs.* 1/T) for mild steel corrosion in 0.5 M HCl in the absence and presence of different concentrations of (**a**) SS; (**b**) AM; (**c**) AR; (**d**) TZ; and (**e**) FG; without KI (**left-hand side**) and with KI (**right-hand side**).

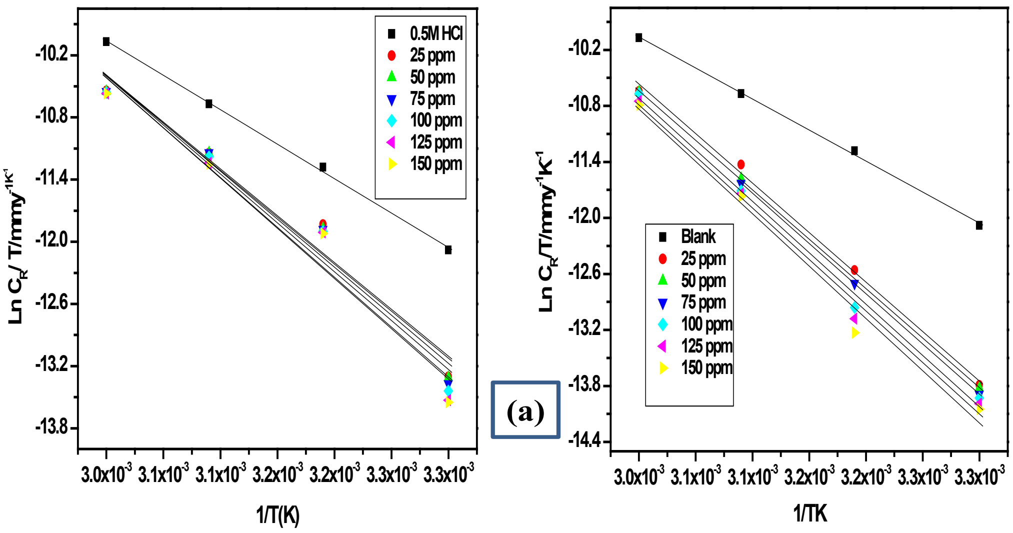

Figure S2. *Cont.*

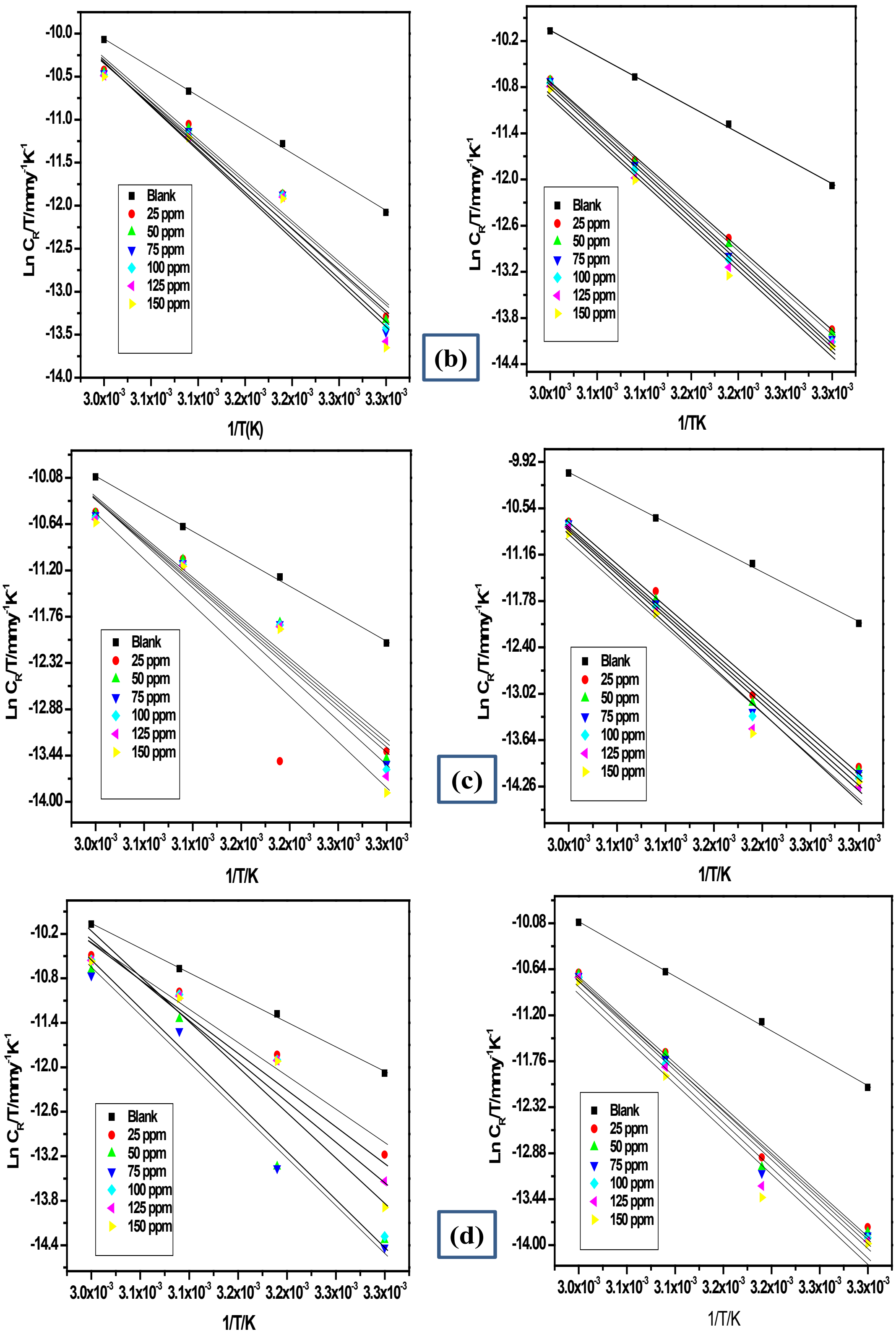

Figure S2. *Cont.*

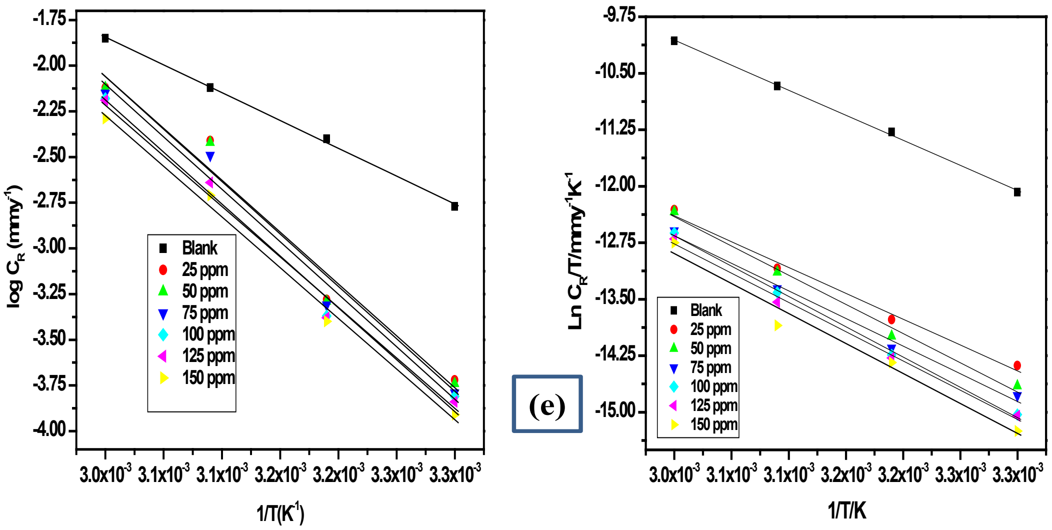

Figures S2. The transition state plots (log CR/T *vs.* 1/T) for mild steel corrosion in 0.5 M HCl in the absence and presence of different concentrations of (**a**) SS; (**b**) AM; (**c**) AR; (**d**) TZ; and (**e**) FG; without KI (**left hand side**) and with KI (**right hand side**).

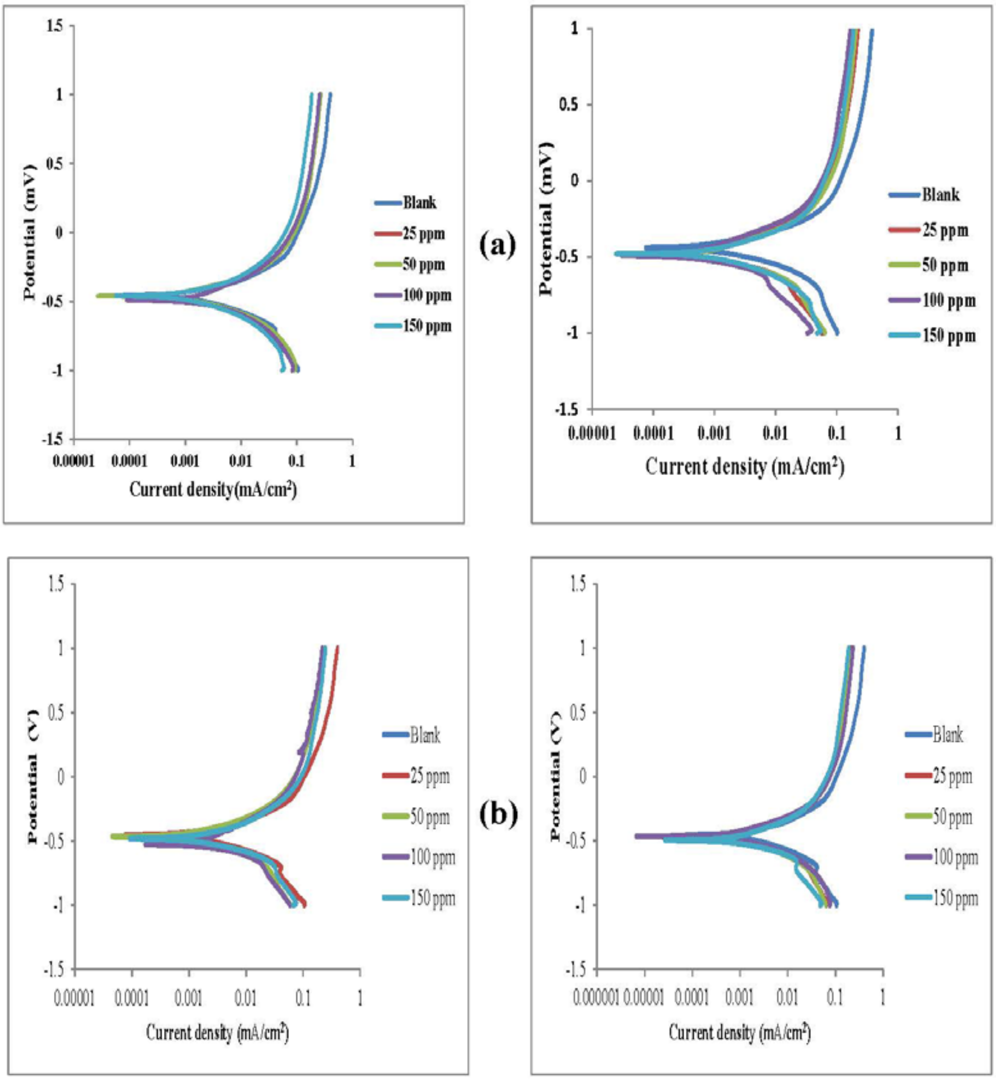

Figure S3. *Cont.*

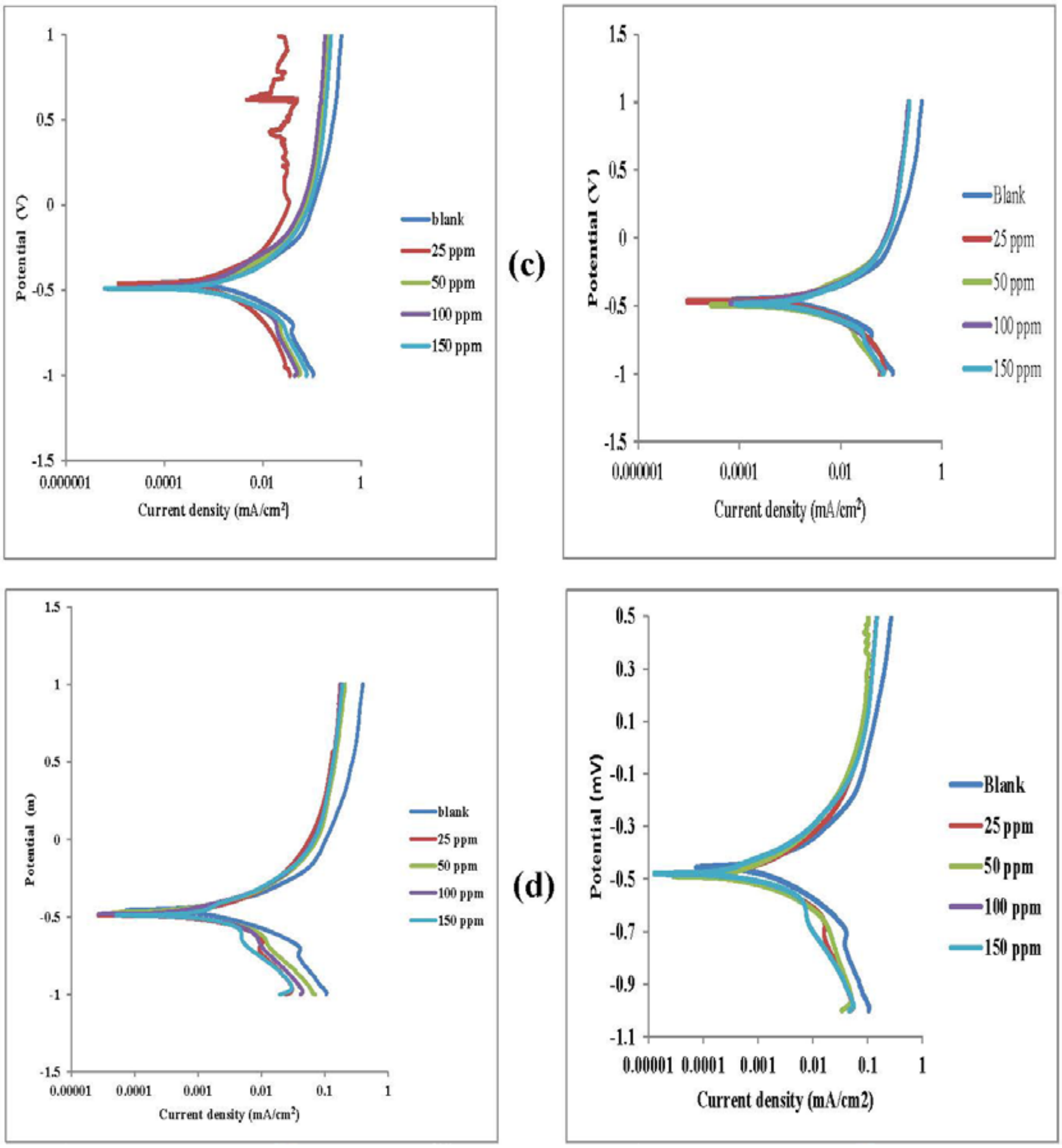

Figure S3. Potentiodynamic polarization curves for mild corrosion in 0.5 M HCl without and with various concentrations of (**a**) AM; (**b**) AR; (**c**) TT; and (**d**) FG; without KI (**left-hand side**) and with KI (**right-hand side**).

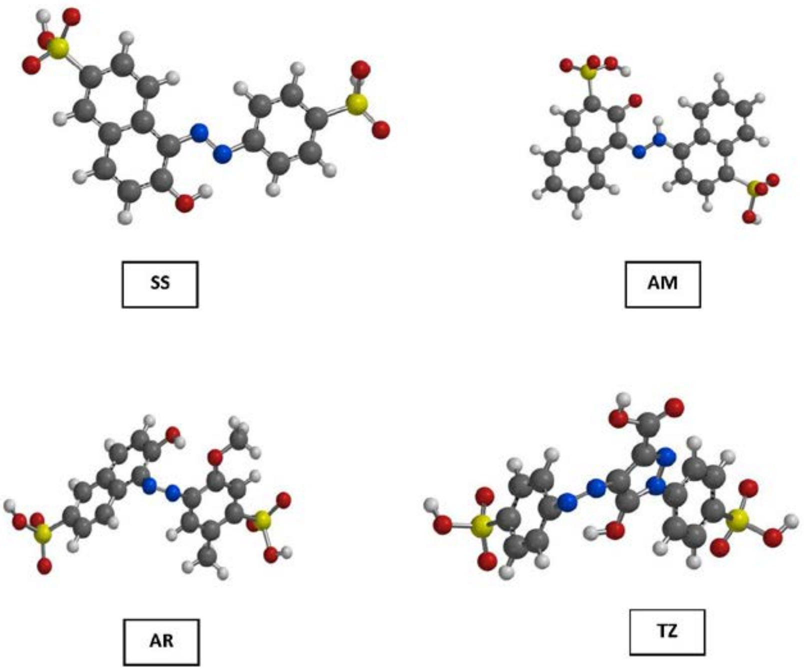

Figure S4. Optimized molecular structures of the studied dyes. Only the lowest energy conformer of each structure is shown. The blue colour represents the N atoms, the red colour representst O atoms, the grey colour represents C atoms, the yellow colour represents S atoms and the while color represent H atoms.

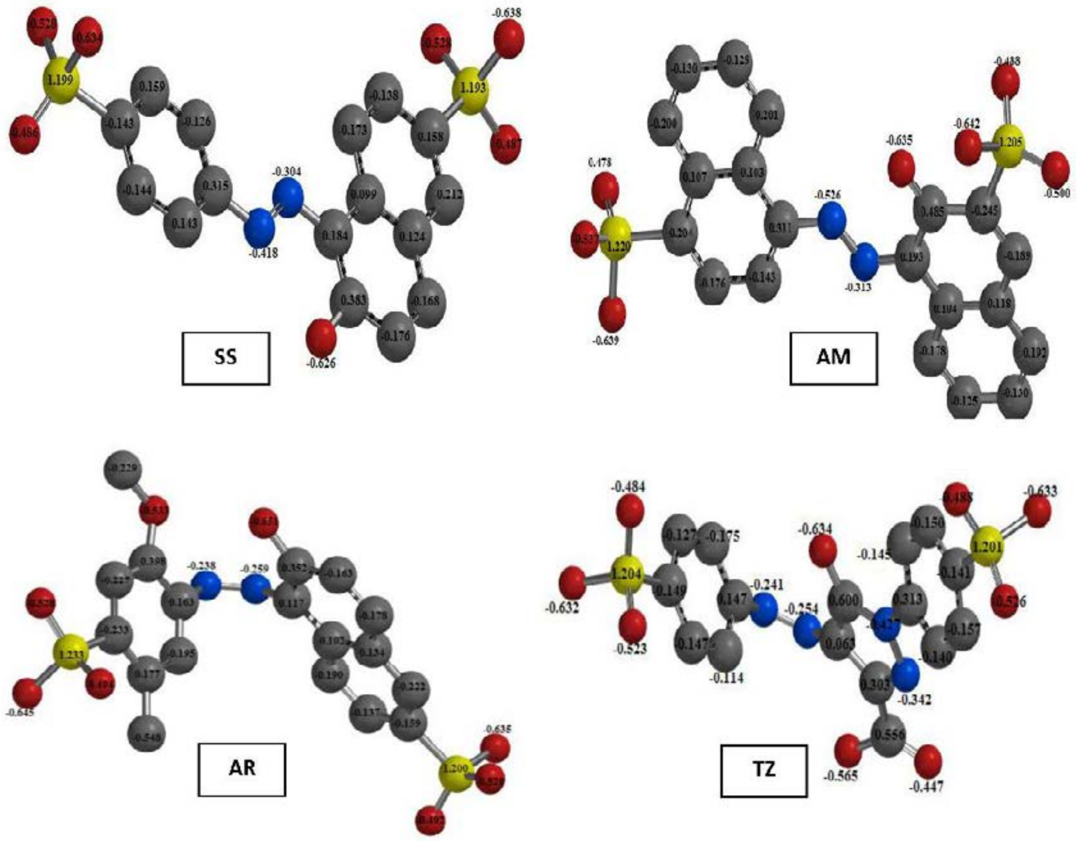

Figure S5. Mulliken atomic charges for the studied dyes.

Table S1. The estimated Fukui indices for the studied food dyes.

SS			AM			AR			TZ		
**Atoms**	f^+	f^-	**Atoms**	f^+	f^-	**Atoms**	f^+	f^-	**Atoms**	f^+	f^-
O1	−0.019	−0.027	O1	−0.027	−0.023	O1	−0.025	−0.022	O1	−0.029	−0.023
S2	−0.022	−0.025	S2	−0.027	−0.028	S2	−0.036	−0.021	S2	−0.040	−0.020
O3	−0.003	−0.007	O3	−0.019	−0.015	O3	−0.022	−0.022	O3	−0.027	−0.020
O4	−0.017	−0.027	O4	−0.024	−0.017	O4	−0.009	−0.010	O4	−0.011	−0.012
C5	−0.009	−0.026	C5	0.020	−0.007	C5	−0.025	−0.022	C5	−0.028	−0.020
C6	−0.019	−0.017	C6	−0.047	−0.017	C6	−0.001	0.000	C6	−0.014	−0.007
C7	−0.003	−0.033	C7	−0.015	−0.045	C7	0.005	0.004	C7	−0.015	−0.012
C8	−0.023	0.004	C8	−0.019	0.003	C8	−0.018	−0.010	C8	−0.001	0.271
C9	0.012	−0.001	C9	0.013	−0.005	C9	0.017	0.021	C9	−0.017	−0.016
C10	−0.016	−0.023	C10	−0.050	−0.020	C10	−0.033	−0.027	C10	−0.011	−0.007
C11	−0.042	−0.031	O11	−0.060	−0.035	C11	−0.011	−0.445	N11	−0.098	−0.100
C12	−0.006	−0.011	C12	−0.012	−0.015	O12	0.008	−0.011	O12	−0.032	−0.028
C13	−0.043	−0.029	C13	−0.011	−0.023	C13	0.022	0.024	N13	−0.114	−0.078
C14	−0.001	−0.064	C14	−0.015	−0.004	N14	−0.116	−0.075	C14	0.035	−0.036
O15	−0.045	−0.059	C15	−0.008	−0.026	N15	−0.111	−0.055	C15	`−0.057	−0.042
N16	−0.080	−0.007	N16	−0.075	0.002	O16	−0.031	−0.050	N16	0.004	−0.011
N17	−0.053	−0.036	N17	−0.019	−0.033	C17	−0.679	−0.030	N17	−0.043	−0.048
C18	−0.002	−0.002	C18	0.001	−0.016	C18	−0.008	−0.006	C18	−0.031	−0.029
C19	−0.015	−0.021	C19	−0.026	−0.035	C19	−0.037	−0.036	C19	−0.027	−0.026
C20	−0.013	−0.007	C20	0.000	−0.007	C20	0.012	0.004	O20	−0.037	−0.042
C21	−0.027	−0.026	C21	−0.029	−0.037	C21	−0.095	−0.004	O21	0.015	0.007

Table S1. *Cont.*

SS			AM			AR			TZ		
**Atoms**	f^{+}	f^{-}	**Atoms**	f^{+}	f^{-}	**Atoms**	f^{+}	f^{-}	**Atoms**	f^{+}	f^{-}
C22	−0.006	−0.008	C22	0.004	0.004	C22	0.012	−0.044	C22	−0.001	−0.003
C23	−0.028	−0.023	C23	−0.015	−0.013	C23	0.004	−0.022	C23	−0.002	−0.011
S24	−0.036	−0.023	C24	−0.002	−0.018	C24	−0.019	−0.018	C24	−0.005	−0.291
O25	−0.027	−0.027	C25	−0.010	−0.017	C25	−0.006	−0.018	C25	−0.013	−0.020
O26	0.001	−0.021	C26	−0.006	−0.012	C26	−0.018	−0.021	C26	−0.007	−0.008
O27	−0.025	−0.023	C27	−0.010	−0.021	S27	−0.024	−0.022	C27	−0.002	−0.013
			S28	−0.029	−0.026	O28	−0.008	−0.012	S28	−0.020	−0.020
			O29	−0.006	−0.010	O29	−0.017	−0.023	O29	−0.024	−0.024
			O30	−0.020	−0.030	O30	−0.018	−0.026	O30	−0.006	−0.012
			O31	−0.021	−0.032				O31	−0.016	−0.022
